# Single Dose Administration of Taheebo Polyphenol Enhances Endurance Capacity in Mice

**DOI:** 10.1038/s41598-018-33029-2

**Published:** 2018-10-02

**Authors:** Koichi Yada, Katsuhiko Suzuki, Natsumi Oginome, Sihui Ma, Youichi Fukuda, Akira Iida, Zsolt Radak

**Affiliations:** 1 Faculty of Sport Sciences, Waseda University, Tokorozawa, Saitama Japan; 2 Graduate School of Sports Sciences, Waseda University, Tokorozawa, Saitama Japan; 3 Faculty of Agriculture, Kindai University, Naka-machi, Nara Japan; 4 Research Institute of Sport Science, University of Physical Education, Budapest, Hungary

## Abstract

Endurance capacity is important for maintenance of quality of life as well as performance of endurance athletes. In order to improve endurance, intake of nutritional supplements as well as exercise training is also important. Indeed, polyphenolic extracts from plants are known to improve endurance capacity via increase of fatty acid utilization, mitochondrial biogenesis or inhibition of oxidative stress. Taheebo, the extract obtained from inner bark of *Tabebuia avellanedae* has been reported to have beneficial effects for treatment of inflammation, oxidative stress and obesity. Here, we investigated the effects and mechanisms of polyphenol fraction of taheebo (taheebo polyphenol; TP) on endurance capacity of mice. Single dose administration of TP significantly increased running time until exhaustion. Acute TP administration increased blood glucose and muscle glycogen levels (p < 0.05) through alteration on expression level of genes involved with glycogen metabolism and gluconeogenesis. Furthermore, TP administration decreased exercise-induced increase of protein carbonyls in skeletal muscle. These results suggest that TP administration improve endurance capacity via up-regulation of skeletal muscle glycogen levels and maintenance of blood glucose by acceleration of gluconeogenesis as well as inhibition of exercise-induced oxidative stress. Single administration of TP also increased phosphorylation of AMP-activated protein kinase (AMPK) and gene expression level of sirtuin 1 (SIRT1) but did not change the marker of mitochondrial biogenesis.

## Introduction

Endurance capacity is important for not only the performance of the endurance athletes but also maintenance of quality of life. Indeed, it has been reported that high-endurance capacity is associated with lower blood pressure, visceral adipose fat, plasma triglyceride, insulin and glucose levels compared with low-endurance capacity^1^ and longer life span in rats^2^. The main sources of energy during endurance exercises are carbohydrate (CHO) and lipids. During low-intensity prolonged exercise, fat is the main source for ATP synthesis. However, demand of CHO utilization increases as exercise intensity increases. It is known that exercise-induced exhaustion can occur when muscle glycogen is almost depleted, especially when exercise is done with moderate intensity and long duration^3,4^. The level of glycogen stores and the ability to increase glycogen stores are crucial limiting factors for aerobic endurance. On the other hand, when exercise is done with high intensity, the reason of fatigue is due to the accumulation of different metabolites, such as lactic acid, Pi, ammonia or Ca^++^ ^5–8^.

It is well known that exercise training and intake of nutritional supplement are both important strategies for the improvement of endurance capacity. Various studies have shown that several nutritional supplements such as green tea extracts, polyphenols, L-carnitine, taurine and capsaicin could improve endurance capacity^9^. These supplements enhance the endurance capacity via several mechanisms. For example, green tea extracts increase running time via enhancement of fatty acid oxidation^10,11^. Quercetin increases endurance performance via activation and/or up-regulation of peroxisome proliferator-activated receptor gamma coactivator-1 alpha (PGC-1α) and mitochondrial biogenesis^12^. Other polyphenols have also been reported to inhibit oxidative stress in several conditions such as obesity, strenuous exercise and ultraviolet radiation^13–18^. Most of these supplementations were chronic suggesting along with training protocols, whereas the acute effects are rarely studies.

Since oxidative stress is one of the main fatigue mediators during exercise^19^, polyphenols may improve the endurance capacity via not only the enhancement of substrate oxidation but also inhibition of oxidative stress.

*Tabebuia avellanedae* is a tree native to the tropical rain forest from Brazil to North Argentina in South America. The extract obtained from inner bark of *Tabebuia avellanedae* is called taheebo. Taheebo has been traditionally used for the treatment of several diseases in South America. Recently, taheebo has been reported to have beneficial effects for treatment of cancer, inflammation, oxidative stress and obesity^20–23^. However, it is unclear whether the signaling pathways that are modulated by taheebo extracts can contribute to enhancement of endurance capacity. Therefore, the aim of the present study was to investigate the effects of taheebo supplementation on endurance capacity using the polyphenol fraction (taheebo polyphenol; TP).

## Materials and Methods

### Animals

Seventy-eight male C57BL/6 J mice (8 weeks old) were purchased from Takasugi experimental animals supply (Kasukabe, Japan). Four or five animals were housed together in 1 cage (27 × 17 × 13 cm) in a controlled environment under a light-dark cycle (lights on at 0900 and off at 2100). The experimental procedures followed the Guiding Principles for the Care and Use of Animals in the Waseda University Institutional Animal Care and Use Committee and were approved by the Institutional Animal Care and Use Committee in the university.

### Extraction of polyphenol fraction of Tabebuia avellanedae

The dried inner bark of *Tabebuia avellanedae* (2.0 kg), which was generously provided by Taheebo Japan Co., Ltd (Osaka, Japan) was extracted using with boiling water (12.8 L) two times for 1 h. The water solution was subjected to polyamide (400 g) column chromatography and eluted by water (6.0 L), 30% MeOH aq. (1.5 L), 40% MeOH aq. (1.5L), 50% MeOH aq. (2.5 L) and 100% MeOH. The 50% MeOH aq. elution was concentrated in vacuo. The residue (12.6 g) was followed by silicagel column chromatography and eluted by CHCl_3_ - MeOH - H_2_O (14: 6: 1). Then, we used the concentrated fraction (7.7 g) which exhibited coloration in a dark green color by a mist of the ferric chloride reagent on thin-layer chromatography (TLC; Silica gel G) from the eluent and is demonstrated to be polyphenols especially acteoside as the main constituent.

### Experimental protocol

One week before the exhaustive exercise, all mice were once accustomed to treadmill running at 15 m/min for 10 min. On the day of the experiment, all mice were randomly assigned to one of the four groups: control group (Con; water administrated + rest, n = 14), TP administrated group (TP; TP administrated + rest, n = 14), exercise group (Ex; water administrated + exercise, n = 25) or TP administrated plus exercise group (TP + Ex; TP supplemented + exercise, n = 25). One hour before the exhaustive exercise on a motorized treadmill (Natsume, Kyoto, Japan), mice in TP and TP + Ex groups were given TP (200 mg/kg weight) with a feeding needle. Mice in the Con and Ex groups were given water. Then, mice in the Ex and TP + Ex groups were subjected treadmill running at 10 m/min for 15 min, followed by 15 m/min and 20 m/min for 15 min each, and then 24 m/min and 7% grade until exhaustion. The exhaustion was defined as the inability to continue regular treadmill running despite the stimulation of repeated tapping on the back of the mouse. The running time of exercised mice were recorded.

Immediately and 4 h after the exhaustion, mice (Con and TP group mice, n = 7, Ex and TP + Ex group mice, n = 8, respectively) were sacrificed under light anesthesia with the inhalant isoflurane (Abbott, Tokyo, Japan). We have selected 4 h time period to sacrifice our animals after exhaustive running, because this time could be enough to measure adaptive response to exercise by even increased protein synthesis of proteins with short half-life^24^.

Blood sample was taken using heparin from the abdominal aorta under inhalant isoflurane-induced mild anesthesia and the gastrocnemius muscle and liver were immediately excised and frozen in liquid nitrogen. Plasma was obtained from blood samples by centrifugation at 1600 g for 10 min at 4 °C. These samples were stored at −80 °C until analyses. For the measurement of glycogen, gastrocnemius muscle and liver were homogenized in distilled water. For the measurement of tissue glycerol, skeletal muscle and liver were homogenized in Glycerol Assay Buffer contained in the assay kit. Then, the homogenate was centrifuged at 10000xg for 5 min at 4 °C and the supernatant was used. For the measurements of antioxidant enzymes activities, protein carbonyl, and immuno blot analysis, skeletal muscle was homogenized in tissue protein extraction reagent (T-PER; Pierce, Rocford, IL) containing protease inhibitor (Complete mini protease inhibitor cocktail tablet; Roche Diagnostics, Mannheim, Germany) and phosphatase inhibitor (Roche). Then, the homogenate was centrifuged at 10000xg for 15 min at 4 °C and the supernatant was used. For the measurements of glutathione and trolox equivalent antioxidant capacity (TEAC), skeletal muscle was homogenized in phosphate buffered saline (PBS; pH 7.4).

### Measurement of plasma biochemical parameters, tissue glycogen and markers of oxidative stress

Plasma concentrations of glucose, lactic acid (LA), non-esterified fatty acid (NEFA), triglyceride (TG), blood urea nitrogen (BUN), total ketone body (T-KB), uric acid (UA), aspartate aminotransferase (AST), alanine aminotransferase (ALT), creatine kinase (CK) and lactate dehydrogenase (LDH) were measured by Oriental Yeast Co. (Tokyo, Japan). Plasma glycerol concentration was measured with Glycerol Colorimetric Assay Kit (Cayman Chemical Co., Ann Arbor, MI). Skeletal muscle and liver glycerol levels were measured with Free Glycerol Assay Kit II (BioVison, Inc., Milpitas, CA). Tissue glycogen concentration was measured with Glycogen Assay kit (BioVison, Inc.). Protein carbonyl, a marker of oxidative stress, in muscle was measured with Protein Carbonyl Assay Kit (Cayman Chemical Co.). Superoxide dismutase (SOD), catalase (CAT) and glutathione peroxidase (GPx) were measured with SOD Assay Kit (Cayman Chemical Co.), Catalase Assay Kit (Cayman Chemical Co.) and GPx Assay Kit (Cayman Chemical Co.), respectively. Reduced glutathione (GSH) was determined using a commercially available assay kit (Biooxytech GSH/GSSG-412; Oxis Health Products, Portland, OR). Non-enzymatic antioxidant capacity TEAC in muscle and plasma were measured according to the assay described by Re, *et al*. (2000)^25^.

### Real-time quantitative polymerase chain reaction (PCR)

Total RNA was extracted from the gastrocnemius muscle and liver using the RNeasy Mini Kit (Qiagen, Valencia, CA) according to the manufacturer’s instructions. The purity of total RNA was assessed using the NanoDrop system (NanoDrop Technologies, Wilmington, DE). Total RNA was reverse transcribed to cDNA using the High Capacity cDNA Reverse Transcription Kit (Applied Biosystems, Foster City, CA) according to the manufacturer’s instructions. PCR was performed with the Fast 7500 real-time PCR system (Applied Biosystems) using the Fast SYBR^®^ Green PCR Master Mix (Applied Biosystems). The thermal profiles consisted of 10 min at 95 °C for denaturation followed by 40 cycles of 95 °C for 3 s and annealing at 60 °C for 15 s. 18 s mRNA was used as the housekeeping gene, and the ΔΔCT method was used as previously described [30] to quantify target gene expression. All data are represented relative to its expression as fold change based on the values of the Con group. Specific PCR primer pairs for each studied gene are shown in Table 1.

**Table 1:** Primer sequence for Real-Time PCR analysis.

Genes	Forward	Reverse
GS	ACTGCTTGGGCGTTATCTCTGTG	ATGCCCGCTCCATGCGTA
GP	TGGCAGAAGTGGTGAACAATGAC	CCGTGGAGATCTGCTCCGATA
HK2	CTGTCTACAAGAAACATCCCCATTT	CACCGCCGTCACCATAGC
G6Pase	GTGGCAGTGGTCGGAGACT	ACGGGCGTTGTCCAAAC
PEPCK	CACCATCACCTCCTGGAAGA	GGGTGCAGAATCTCGAGTTG
HADH	ACTACATCAAAATGGGCTCTCAG	AGCAGAAATGGAATGCGGACC
MCAD	GCTCGTGAGCACATTGAAAA	CATTGTCCAAAAGCCAAACC
ACO	TGTTAAGAAGAGTGCCACCA	ATCCATCTCTTCATAACCAAATTT
CPT1α	CCAGGCTACAGTGGGACATT	GAACTTGCCCATGTCCTTGT
CPT1β	CCCATGTGCTCCTACCAGAT	CCTTGAAGAAGCGACCTTTG
CPT2	GAAGAAGCTGAGCCCTGATG	GCCATGGTATTTGGAGCACT
ACC1	ATTGGGCACCCCAGAGCTA	CCCGCTCCTTCAACTTGCT
ACC2	GGGCTCCCTGGATGACAAC	TTCCGGGAGGAGTTCTGGA
MCD	ACTCCATCAGCCTGACCCAG	ACCCCTTGAGGCTCTCGTGA
PGC-1α	GACTGGAGGAAGACTAAACGGCCA	GCCAGTCACAGGAGGCATCTTT
SIRT1	GCAACAGCATCTTGCCTGAT	GTGCTACTGGTCTCACTT
Cyto c	CACGCTTTACCCTTCGTTCT	CTCATTTCCCTGCCATTCTC
18S	CGGCTACCACATCCAAGGA	AGCTGGAATTACCGCGGC

GS; glycogen synthase, GP; glycogen phosphorylase, HK2; hexokinase 2, G6Pase; glucose-6-phosphatase, PEPCK; phosphoenolpyruvate carboxykinase, HADH; hydroxyacyl-CoA dehydrogenase, MCAD; medium chain acyl-CoA dehydrogenase, ACO; acyl-CoA oxidase, CPT; carnitine palmitoyltransferase, ACC; acetyl-CoA carboxylase, MCD; malonyl-CoA decarboxylase, PGC-1α; peroxisome proliferator-activated receptor gamma coactivator-1 alpha, SIRT1; sirtuin 1, Cyto c; cytochrome c, 18 S; 18 s ribosomal RNA.

### Western blot analysis

Homogenized samples were diluted with homogenizing buffer to 5 mg/mL protein. Samples were then mixed with 4 × Laemmli sample buffer (Bio-Rad, Richmond, CA) in 10% 2-mercaptoethanol to 4 mg protein/mL and heated at 60 °C for 15 min. Aliquots of samples were separated by electrophoresis on 10% SDS–polyacrylamide gels and then transferred to polyvinyl difluoride (PVDF) membranes. The membranes were blocked for 1 h at room temperature in tris-buffered saline/0.05% Tween 20 (TBST) with 5% skim milk. Then, membranes were incubated overnight in the TBST in 5% BSA containing anti-phospho-AMP-activated protein kinase (AMPK) α Thr172 (#2535; Cell Signaling Technology, Beverly, MA) and anti-AMPKα (#2532; Cell Signaling). The membranes were washed with TBST and incubated with appropriate secondary horseradish peroxidase-conjugated antibodies (1:30000; Bio-Rad), visualized by enhanced chemiluminescence (ECL; GE Healthcare, Arlington Heights, IL) and quantified by densitometry (Las-3000, Fuji photo, Tokyo, Japan).

### Statistical analysis

Data are presented as means ± standard errors (SE). For comparison of means between two groups, Students’ unpaired t-test was performed. A two-way analysis of variance (ANOVA) was performed to determine the main effects of TP administration and/or exercise. Statistical analysis was done using SPSS V22.0 (IBM Japan, Ltd, Tokyo, Japan). When this analysis revealed significant interaction, Bonferroni’s post-hoc test was performed to determine the significance among the means. Statistical significance was defined as p < 0.05.

## Results

### Effect of TP administration on endurance capacity

**Figure 1:**
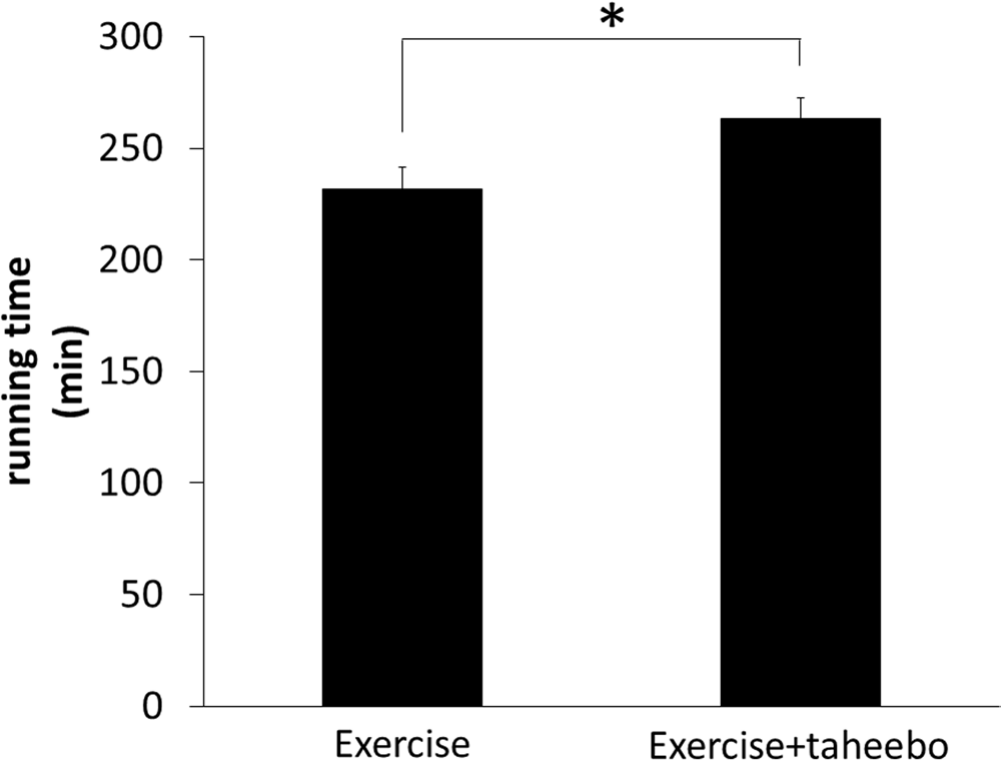
The run time to exhaustion in the Ex group (n = 25) and the TP + Ex group (n = 25). Values are means ± SE. *p < 0.05, compared with the Ex group.

The running time of the mice of the TP + Ex group were significantly higher than that of the mice of the Ex group (263 ± 9.4 min vs 232 ± 9.9 min, p = 0.023, Fig. 1). Thus, single dose of TP administration improved the endurance capacity of mice on treadmill running nearly by 14%.

### Effects of exhaustive exercise and TP administration on plasma glucose level, skeletal muscle and liver glycogen concentrations

**Figure 2:**
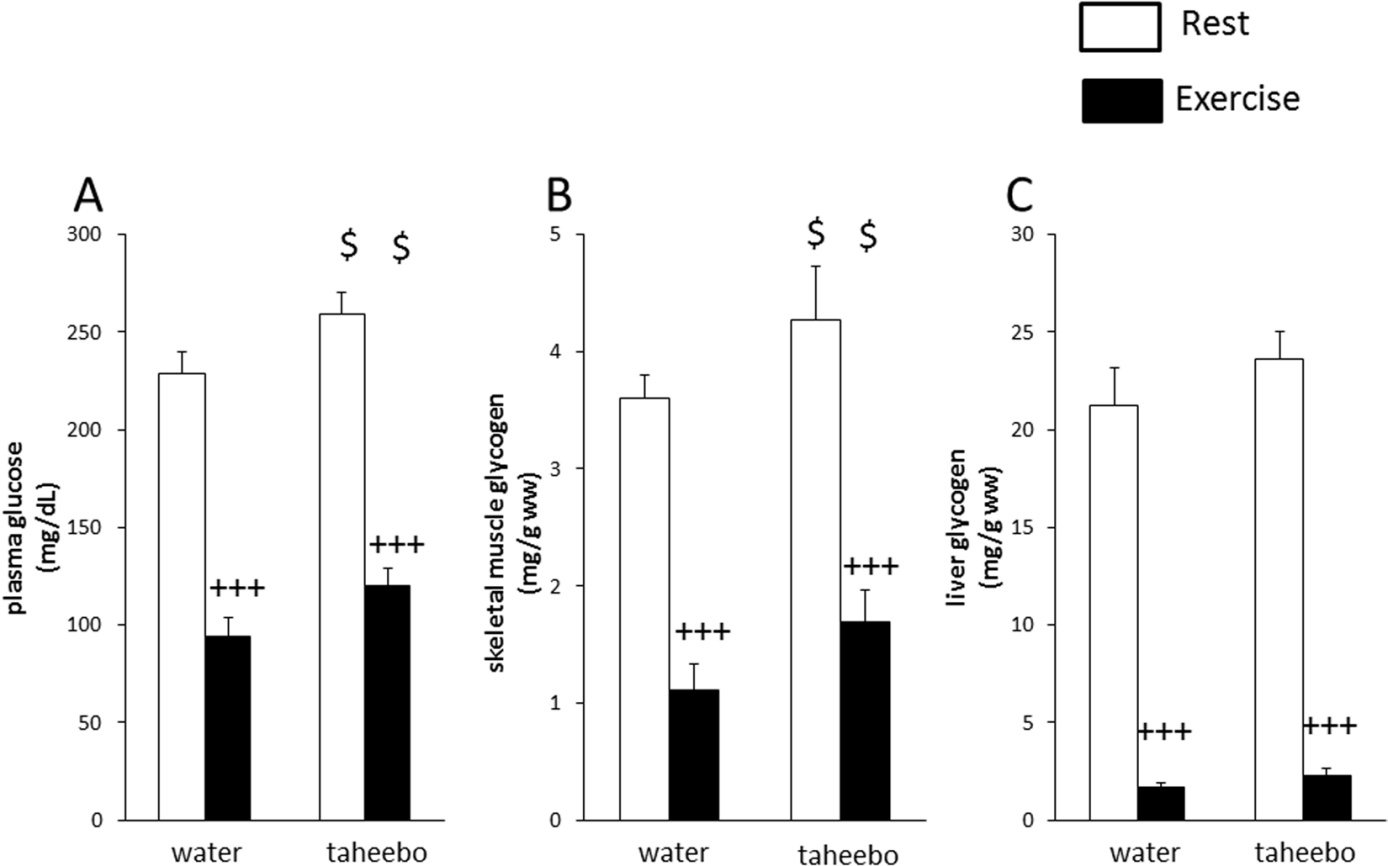
Plasma glucose (**A**), skeletal muscle (**B**) and liver (**C**) glycogen concentrations of the Con (n = 7), TP (n = 7), Ex (n = 8) and TP + Ex (n = 8) groups immediately after the exercise session. Values are means ± SE. ^+++^p < 0.001 main effect for exercise, ^$^p < 0.05 main effect for taheebo polyphenol (TP) administration.

Plasma glucose and skeletal muscle glycogen concentration decreased close to 60% and 70% while liver glycogen concentrations were dropped to lover level than 10% of the initial value as a result exhaustive exercise (p < 0.001, Fig. 2A–C). TP administration increased plasma glucose and glycogen concentrations (13% and 19% respectively) in skeletal muscle (p < 0.05, Fig. 2A,B).

### Effects of exhaustive exercise and TP administration on plasma, skeletal muscle and liver glycerol concentrations

**Figure 3:**
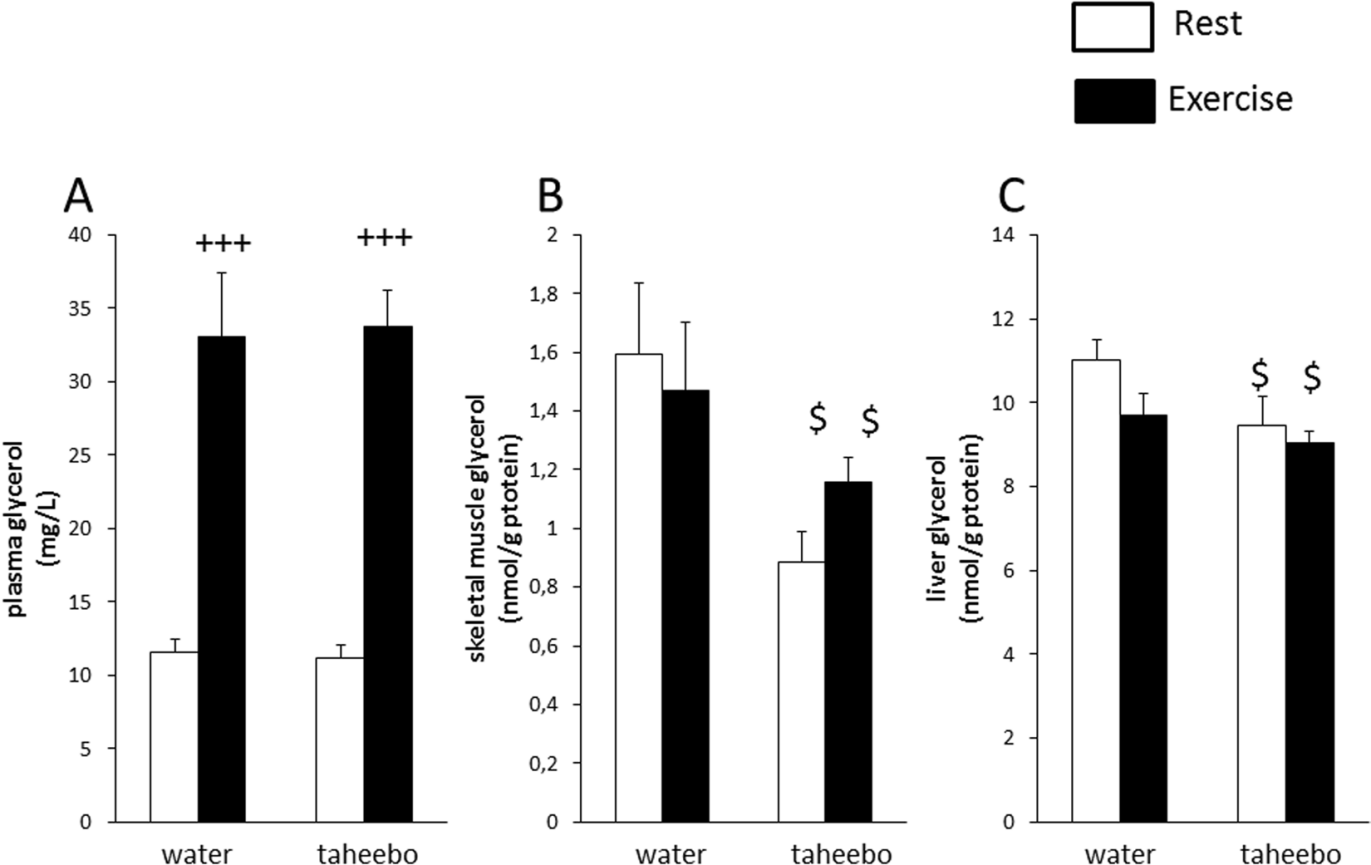
Glycogen synthase (GS) (**A**), glycogen phosphorylase (GP) (**B**) and hexokinase 2 (HK2) (**C**) mRNA expression levels in skeletal muscle of the Con (n = 7), TP (n = 7), Ex (n = 8) and TP + Ex (n = 8) groups immediately after the exercise session. Values are means ± SE. ^+,++,+++^p < 0.05, p < 0.01, respectively, and p < 0.001 main effect for exercise, ^$^p < 0.05 main effect for taheebo polyphenol (TP) administration.

Exhaustive exercise increased plasma glycerol concentration by 3 fold (Fig. 3A). On the other hand, skeletal muscle and liver glycerol concentrations were decreased by TP administration by 44% and 14% respectively (p < 0.05, Fig. 3B,C).

### Effects of exhaustive exercise and TP administration on blood biomarkers

Exhaustive exercise increased NEFA, BUN and T-KB levels and reduced LA concentration in plasma (p < 0.001, Table 2). One of the interesting findings of this study were that the lactic acid levels of decreased after exhaustive exercise, which could be due to enhanced utilization of LA as an energy source.

**Table 2:** Plasma biochemistry data.

	Con	Ex	TP	TP + Ex
LA (mg/dL)	49.5 ± 6.2	25.3 ± 3.8^+++^	44.2 ± 7.3	26.0 ± 5.3^+++^
NEFA (μEq/L)	641 ± 118	1477 ± 130^+++^	695 ± 135	1492 ± 74^+++^
TG (mg/dL)	56.3 ± 8.2	67.8 ± 6.3	67.7 ± 11.5	65.4 ± 3.4
BUN (mg/dL)	26.5 ± 1.8	67.8 ± 4.9^+++^	23.3 ± 1.6^+++,$^	56.2 ± 3.0^+++,$^
T-KB (μmol/L)	141 ± 23	3049 ± 92^+++^	197 ± 29	3048 ± 218^+++^
UA (mg/dL)	0.60 ± 0.09	0.89 ± 0.07	0.69 ± 0.11	0.70 ± 0.07
AST (IU/L)	59 ± 2	191 ± 18^+++^	58 ± 6	164 ± 15^+++^
ALT (IU/L)	28.7 ± 8.7	99.3 ± 19.1^+++^	27.0 ± 2.0	71.8 ± 10.0^+++^
CK (IU/L)	143 ± 26	1844 ± 383^+++^	80 ± 13	1363 ± 161^+++^
LDH (IU/L)	158 ± 10	855 ± 126^+++^	173 ± 23	798 ± 72^+++^

Values are means ± SE. ^+++^p < 0.001 main effect for exercise, ^$^p < 0.05 main effect for taheebo polyphenol (TP) supplementation.

LA; lactic acid, NEFA non-esterified fatty acid, TG; triglyceride, BUN; blood urea nitrogen, T-KB; total ketone body, UA; uric acid, AST; aspartate aminotransferase, ALT; alanine aminotransferase, CK; creatine kinase, LDH; lactate dehydrogenase.

Moreover, BUN, a marker of protein degradation, was decreased by TP administration (p < 0.05, Table 2). We measured AST and ALT as markers of liver injury, CK and LDH as markers of muscle injury. These injury markers increased immediately after the exhaustive exercise (p < 0.001, Table 2). However, TP administration did not alter these injury markers (Table 2).

### Effects of exhaustive exercise and TP administration on glycogen metabolism-related gene expression in skeletal muscle

**Figure 4:**
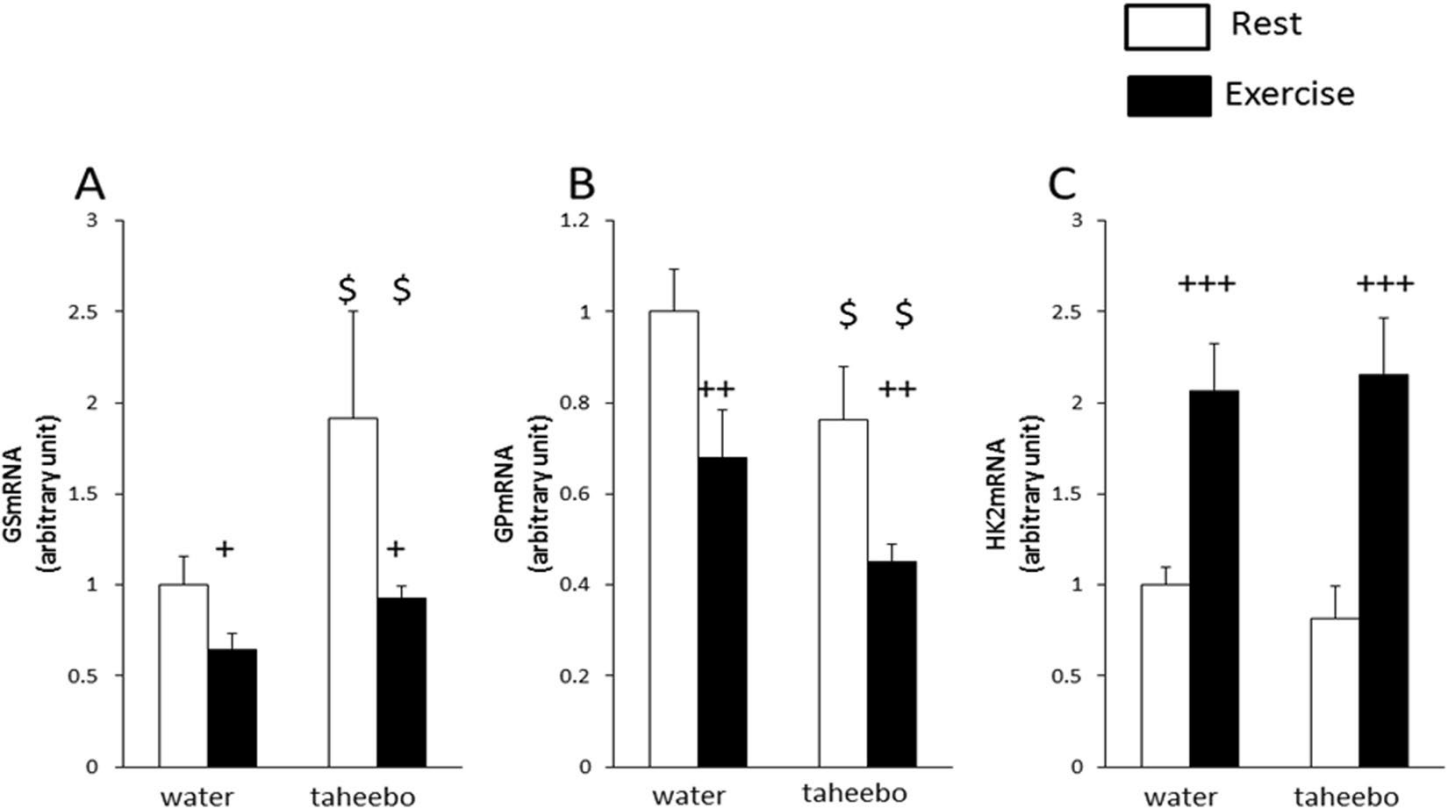
Glycerol concentrations in plasma (**A**), skeletal muscle (**B**) and liver (**C**) of Con (n = 7), TP (n = 7), Ex (n = 8) and TP + Ex (n = 8) groups immediately after the exercise session. Values are means ± SE. ^+++^p < 0.001 main effect for exercise, ^$^p < 0.05 main effect for taheebo polyphenol (TP) administration.

Exhaustive exercise increased hexokinase 2 (HK2) gene expression level and decreased glycogen synthase (GS) and glycogen phosphorylase (GP) gene expression level in skeletal muscle (p < 0.001, p < 0.05 and p < 0.01, respectively, Fig. 4A–C). TP administration increased GS gene expression level and decreased GP gene expression level (p < 0.05, Fig. 4A,B).

### Effects of exhaustive exercise and TP administration on the gene expression level of gluconeogenesis-related genes in liver

**Figure 5:**
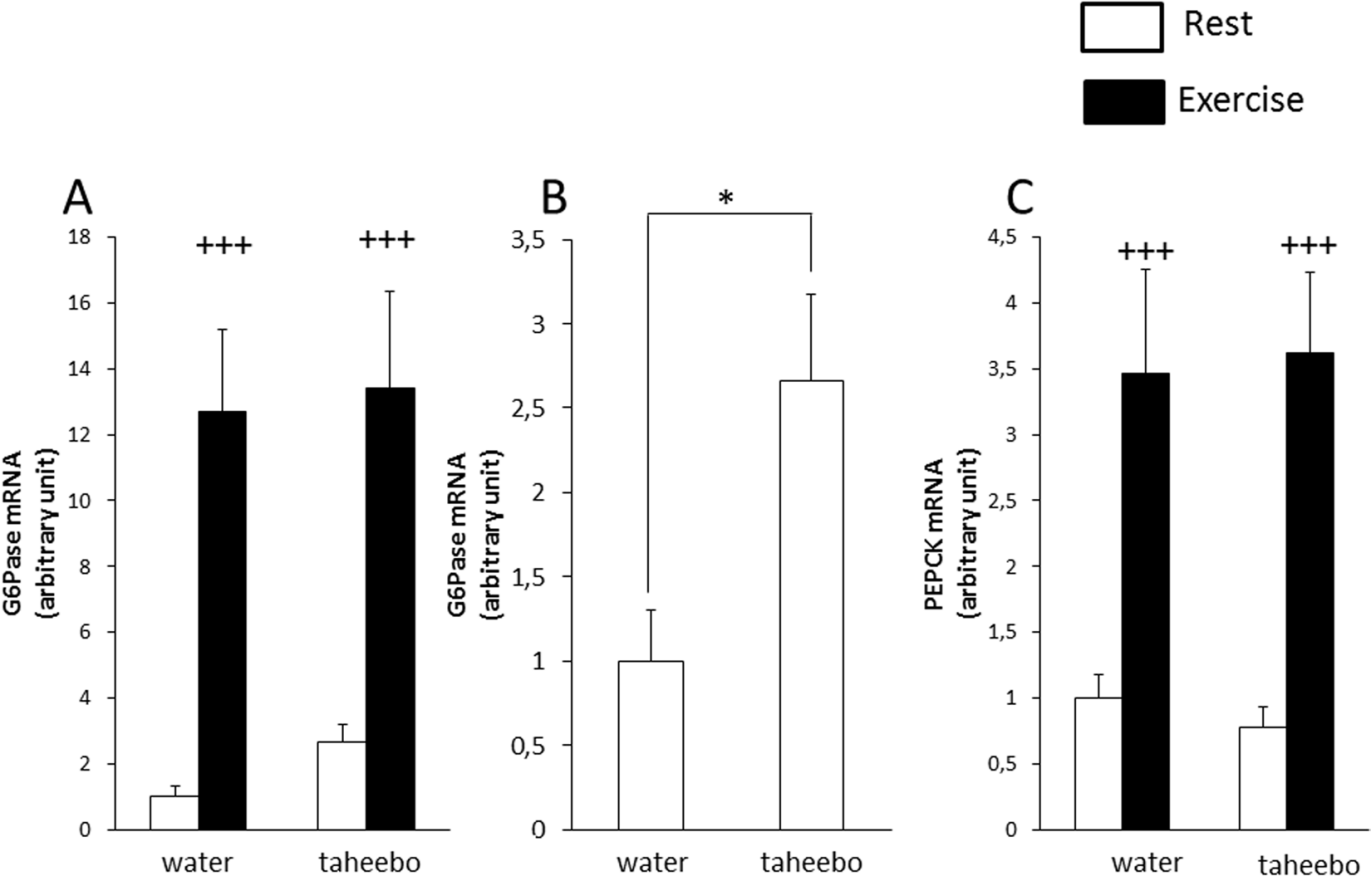
Glucose-6-phospase (G6Pase) mRNA expression levels in skeletal muscle of the Con (n = 7), TP (n = 7), Ex (n = 8) and Ex + TP (n = 8) groups (**A**) and G6Pase mRNA expression level in rest condition mice (Con vs TP) (**B**) and phosphoenolpyruvate carboxykinase (PEPCK) mRNA expression levels in skeletal muscle of the Con (n = 7), TP (n = 7), Ex (n = 8) and TP + Ex (n = 8) groups (**C**) immediately after the exercise session. Values are means ± SE. *p < 0.05, ^+++^p < 0.001 main effect for exercise.

Glucose-6-phosphatase (G6Pase) and phosphoenolpyruvate carboxykinase (PEPCK) gene expression levels in liver were increased by exhaustive exercise (p < 0.001, Fig. 5A,C). Moreover, in the rest condition, liver G6Pase gene expression level of TP administrated group mice was higher compared with that of control group mice (p < 0.05, Fig. 5B).

### Effects of exhaustive exercise and TP administration on the gene expression of fatty acid metabolism-related genes in skeletal muscle

**Figure 6:**
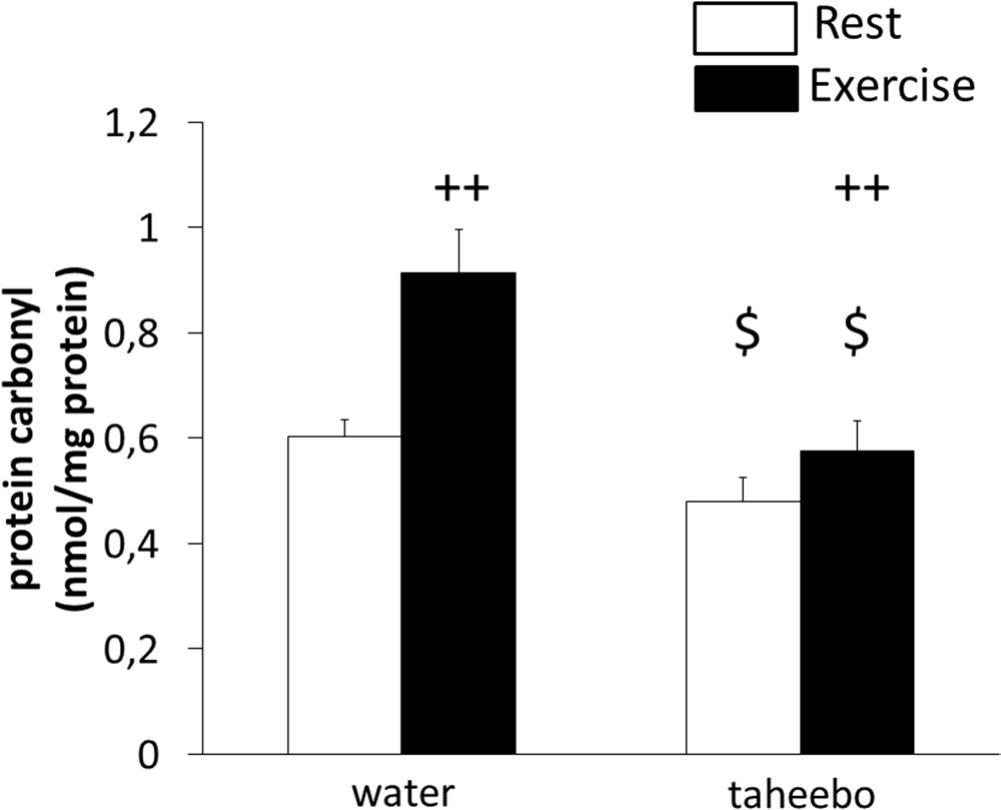
Expression of fatty acid metabolism-rerated genes in skeletal muscle of Con (n = 7), TP (n = 7), Ex (n = 8) and TP + Ex (n = 8) groups immediately after the exercise session. Values are means ± SE. ^++^p < 0.01 main effect for exercise, ^$^p < 0.05 main effect for taheebo polyphenol (TP) administration.

We measured the gene expression of lipid oxidation and fatty acid metabolism-rerated genes to determine the effect of TP on fatty acid metabolism. Gene expression of hydroxyacyl-CoA dehydrogenase (HADH) and medium chain acyl-CoA dehydrogenase (MCAD), which are β-oxidation-related genes, decreased by exhaustive exercise (p < 0.01, Fig. 6A,B). However, acyl-CoA oxidase (ACO), which is another β-oxidation-related gene, was not altered by exercise (Fig. 6C). However, TP administration did not alter these gene expression levels (Fig. 6A–C). Gene expression levels of carnitine palmitoyltransferase (CPT) 1α, β and CPT2, which relates with the import of fatty acyl-CoA into mitochondria, were not altered by exercise and TP administration (Fig. 6D–F). Acyl-CoA carboxylase (ACC) is an enzyme that produces malonyl-CoA from acyl-CoA. Also, malonyl-CoA is known as inhibitor of CPT1. The gene expression levels of ACC 1 and 2 were not altered by exercise (Fig. 6G,H). ACC 1 was decreased by TP administration (p < 0.05, Fig. 6G). Malonyl-CoA decarboxylase (MCD) is an enzyme that carboxylates malonyl-CoA and produces acetyl-CoA. Gene expression of MCD was not changed by exercise and TP administration (Fig. 6I).

### Effects of exhaustive exercise and TP administration on oxidative stress in skeletal muscle

**Figure 7:**
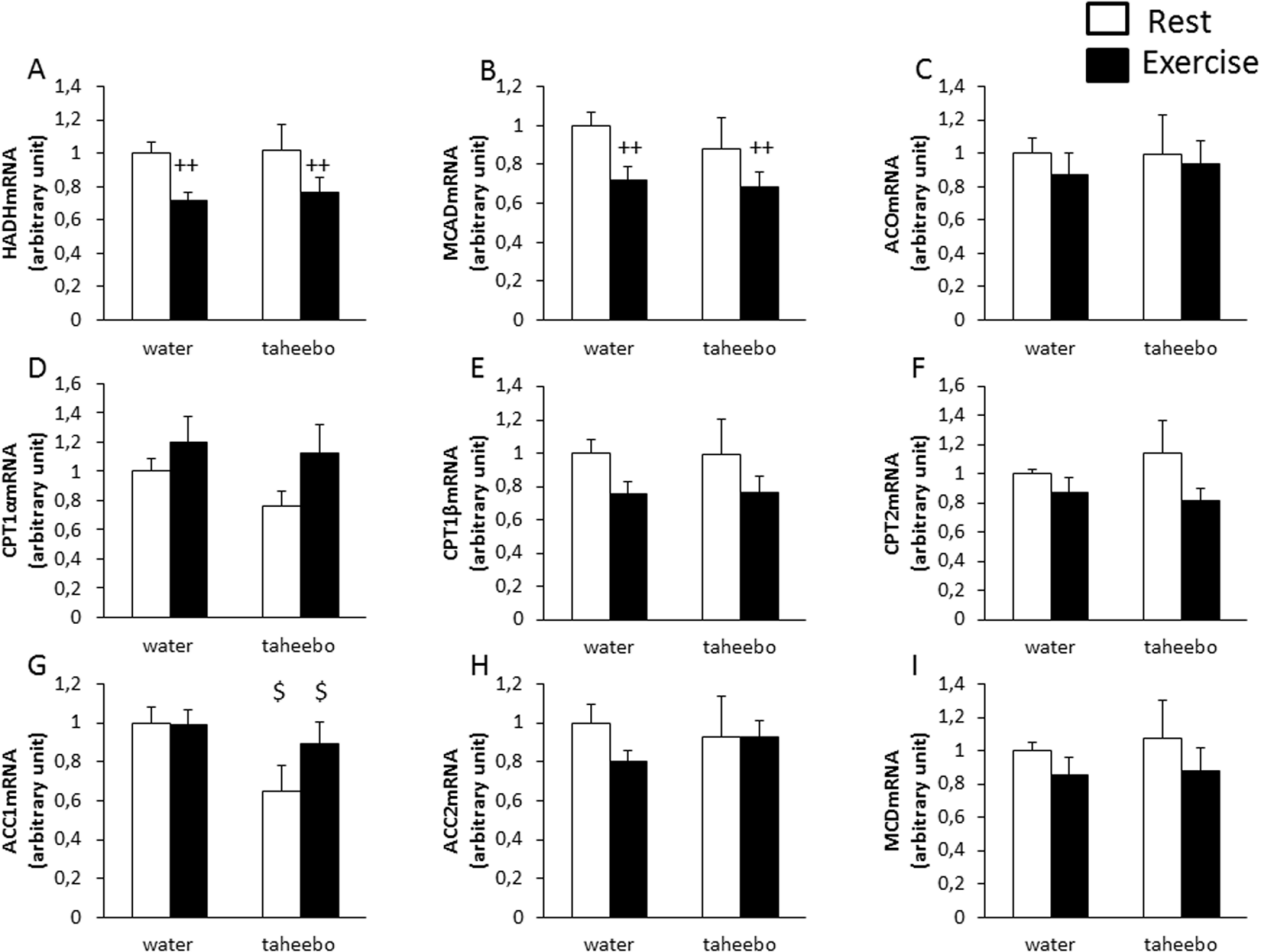
Protein carbonyl levels in skeletal muscle of the Con (n = 7), TP (n = 7), Ex (n = 8) and TP + Ex (n = 8) groups immediately after the exercise session. Values are means ± SE. ^++^p < 0.01 main effect for exercise, ^$^p < 0.05 main effect for taheebo polyphenol (TP) administration.

Protein carbonyl level in skeletal muscle was increased by exhaustive exercise (p < 0.01, Fig. 7). However, TP administration reduced protein carbonyl level in skeletal muscle (p < 0.05, Fig. 7).

### Effects of exhaustive exercise and TP administration on markers of antioxidant capacity

Exhaustive exercise increased GPx activity and TEAC in skeletal muscle and decreased plasma level of TEAC (p < 0.001, p < 0.05 and p < 0.001 respectively, Table 3). On the other hand, TP administration did not affect these antioxidant markers (Table 3).

**Table 3:** Markers of antioxidant in skeletal muscle and plasma.

	Con	EX	TP	TP + EX
SOD activity (U/mg protein)	0.49 ± 0.02	0.41 ± 0.03	0.42 ± 0.02	0.39 ± 0.02
GPx activity (mmol/mg protein)	51.8 ± 1.1	55.2 ± 0.7^+++^	52.3 ± 0.3	55.2 ± 0.4^+++^
CAT activity (μmol/g protein)	22.5 ± 2.6	29.3 ± 3.0	26.2 ± 3.3	24.8 ± 1.3
GSH (μmol/g protein)	3.05 ± 0.14	3.02 ± 0.12	3.07 ± 0.22	2.94 ± 0.12
TEAC (μmol Trolox Eq/g protein)	129.9 ± 3.6	136.1 ± 2.1^+^	131.6 ± 2.5	140.7 ± 3.1^+^
Plasma TEAC (μmol Trolox Eq/mL)	0.84 ± 0.01	0.76 ± 0.02^+++^	0.85 ± 0.01	0.77 ± 0.01^+++^

Values are means ± SE. ^+, +++^p < 0.05, p < 0.001 main effect for exercise.

SOD; superoxide dismutase, GPx; glutathione peroxidase, CAT; catalase, GSH; glutathione, TEAC; Trolox equivalent antioxidant capacity.

### Effects of exhaustive exercise and TP administration on phosphorylation of AMPK in skeletal muscle

**Figure 8:**
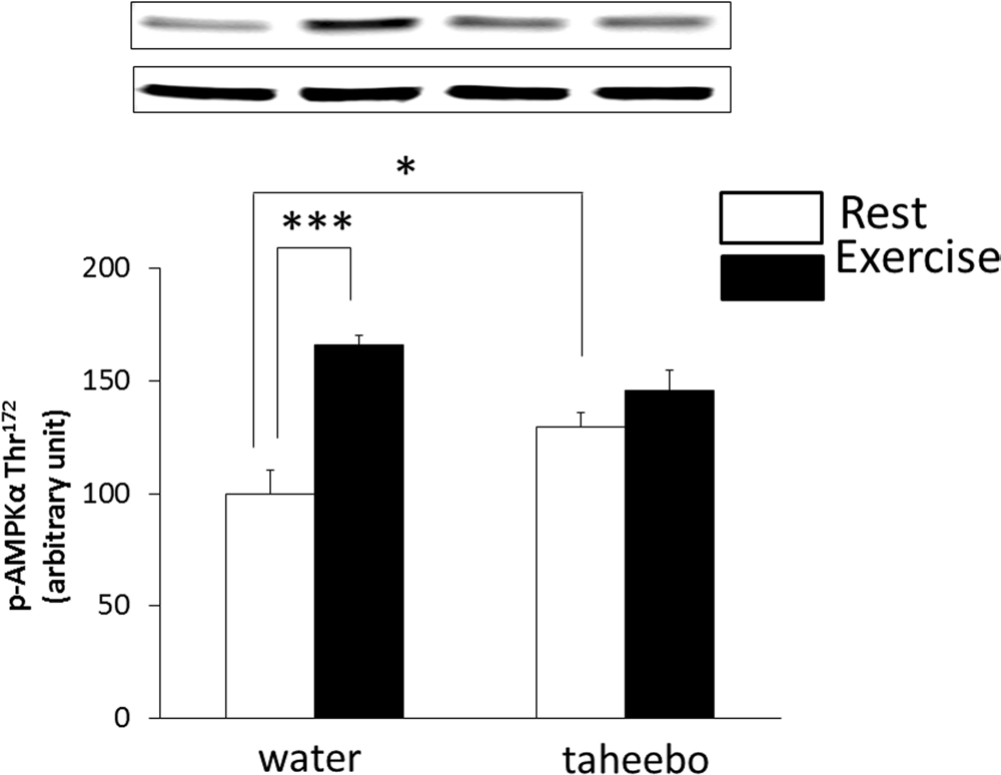
Phosphorylation of AMP-activated protein kinase (AMPK) in the skeletal muscle of the Con (n = 7), TP (n = 7), Ex (n = 7) and TP + Ex (n = 7) groups immediately after the exercise session. Taheboo administration attenuated the exercise-induced metabolic challenge to skeletal muscle. Values are means ± SE. *^,^***p < 0.05, p < 0.001.

The phosphorylation of AMPK in skeletal muscle of Ex group and TP group mice were significantly higher compared with that of Con group mice (p < 0.001 and p < 0.05, respectively, Fig. 8). However, there was no significant difference of AMPK phosphorylation in skeletal muscle between the TP + Ex group, Ex group, or TP group (Fig. 8).

### Effects of exhaustive exercise and TP administration on gene expression level of skeletal muscle adaptation-related genes

**Figure 9:**
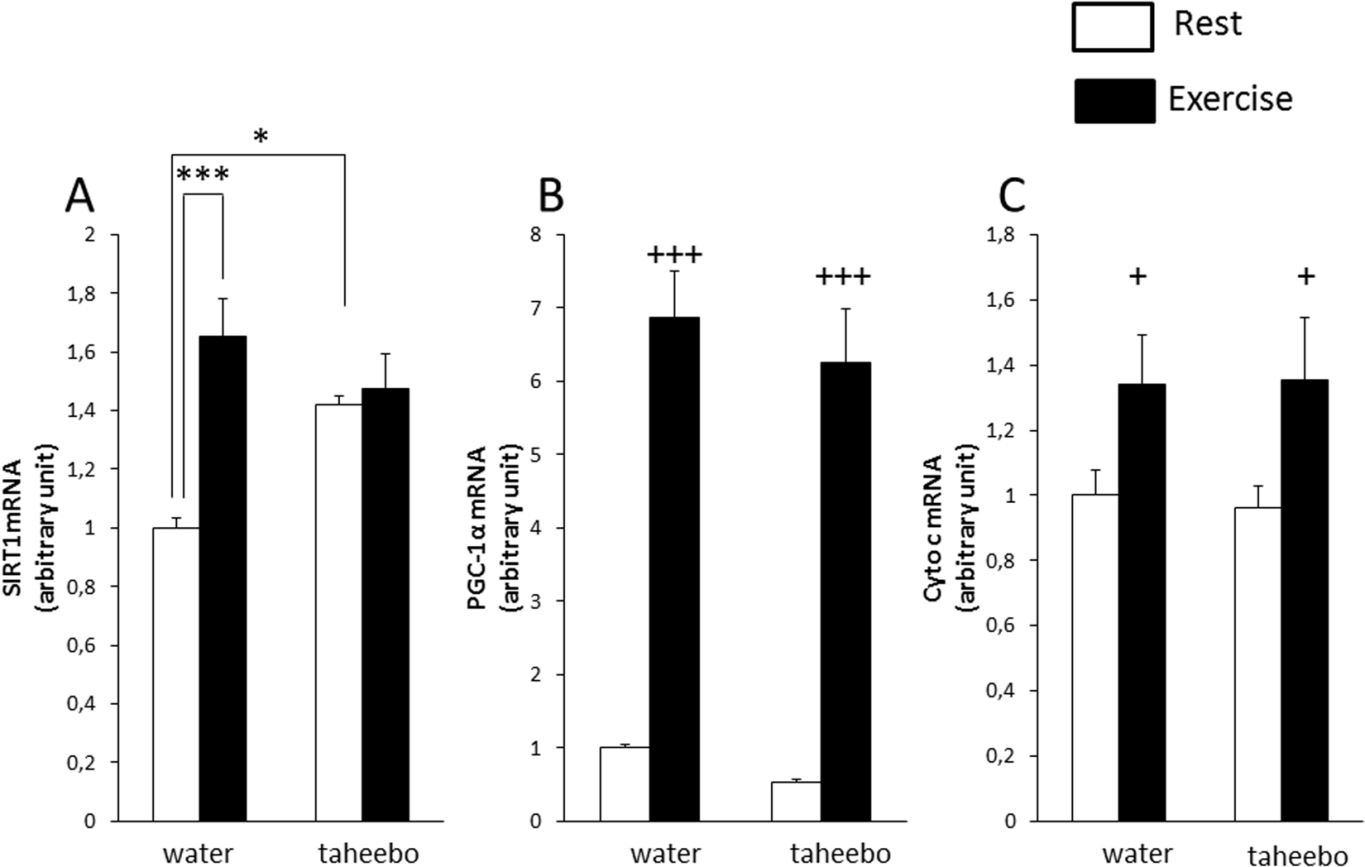
Gene expression of sirtuin 1 (SIRT1) (**A**), peroxisome proliferator-activated receptor gamma coactivator-1alpha (PGC-1α) (**B**), and cytochrome c (Cyto c) (**C**) of the Con (n = 7), TP (n = 7), Ex (n = 8) and TP + Ex (n = 8) groups four hours after the exercise session. Values are means ± SE. *, ***p < 0.05, p < 0.001. ^+,+++^p < 0.05, p < 0.001 main effect for exercise.

We measured gene expression of some key proteins that play an important role in adaptive response to endurance exercise in skeletal muscle samples which were obtained 4 h after the end of the exhaustive exercise. The gene expression of sirtuin (SIRT) 1 in skeletal muscle of the Ex group and TP group were significantly higher compared with the Con group (p < 0.001 and p < 0.05 respectively, Fig. 9A). However, there was no significant difference between the expression of SIRT1 in skeletal muscle of the Ex + TP group and Ex group or TP group (Fig. 9A). The gene expression levels of PGC-1α and cytochrome c (Cyto c) were increased by exhaustive exercise (p < 0.001 and p < 0.05, respectively, Fig. 9B,C). However, these gene expression levels were not affected by TP supplementation (Fig. 9B,C).

## Discussion

In the present study, we investigated the effects of TP on endurance capacity and the related molecular factors. The main finding of the present study is that TP administration enhanced endurance capacity via up-regulation of muscle glycogen levels. Several studies which investigated the effects of taheebo extract have reported that taheebo has anti-cancer, anti-obesity, anti-inflammation and antioxidant effects^20–23,26^. The present study is the first report that lights up the mechanisms by which TP benefits endurance capacity.

In this study, a single dose of TP administration improved endurance capacity, up-regulated blood glucose and muscle glycogen levels and decreased BUN concentration. Aerobic exercise-induced exhaustion is caused by the depletion of muscle glycogen store^3,4^ which induces protein degradation and increases BUN level. BUN is known as the marker of kidney injury as well as the indicator of exercise tolerance^27^. TP supplementation also decreased skeletal muscle and liver concentrations of glycerol, which is one of the sources of hepatic gluconeogenesis. Gluconeogenesis is an important pathway to produce glucose from lactate, amino acid and glycerol in liver or kidney. During fasting or exercise, blood glucose level is maintained through the increase of gluconeogenesis or fat utilization. Therefore, it is suggested that endurance capacity was increased by TP via up-regulation of muscle glycogen and blood glucose levels through acceleration of gluconeogenesis particularly from glycerol and lactate. Glycogen synthesis and degradation are regulated by GS and GP. In this study, gene expression of GS was increased whereas gene expression of GP was decreased by TP. It has been reported that green tea polyphenol increased GS expression in murine liver cells^28^. Furthermore, Kamiyama *et al*. (2010) reported that catechin gallates from green tea decreased GP expression in *in vitro* study^29^. Thus, polyphenols could regulate glycogen synthesis. Therefore, it is considered that TP supplementation-induced accumulation of muscle glycogen could be mediated by up-regulation of GS and down-regulation of GP. Moreover, we measured gene expression of gluconeogenesis-related enzymes because glycerol, a substrate for gluconeogenesis, was decreased with TP administration in this study. TP increased in G6Pase gene expression in rest condition. G6Pase, which removes phosphate from glucose-6-phospate and generates glucose, is one of the key enzymes in gluconeogenesis. Therefore, it is suggested that TP decreased the tissues glycerol and increased plasma glucose level through activation of gluconeogenesis. Additionally, TP did not alter plasma NEFA and TG concentrations. Moreover, we measured fatty acid oxidation-related genes. However, TP scarcely affected these genes expression. Consequently, these results suggest that TP directly affected glycogen accumulation and gluconeogenesis without affecting fatty acid metabolism. However, tissue injury markers, ALT, AST, CK and LDH were increased by exhaustive exercise regardless of administration of TP. These results suggested that exercise-induced tissue damage was not related with running time until exhaustion or the leakage of these small molecular size proteins could be due to increased permeability of sarcolemma.

It is known that oxidative stress is also associated with exercise-induced fatigue^19^. This involvement of oxidative stress on exercise-induced fatigue has been appraised by examining the influence of antioxidant supplementation on exercise performance. It has been reported that increased reactive oxygen species (ROS) reduced muscle tension^30,31^. However, there is a bell-shape curve between ROS levels and force generation of skeletal muscle, and large level of ROS leads to decreased muscle function, which is probably the case during exhaustive exercise. In fact, it was reported that some antioxidants could improve endurance performance^32,33^.

Several *in vitro* studies reported that Taheebo extract has antioxidant capacity^23,34,35^. Consistent with those reports, TP supplementation inhibited muscle oxidative stress in this study. However, TP did not affect the activities of antioxidant enzymes or non-enzymatic antioxidant capacity. These results suggested that TP directly reduced the generation of ROS but did not modify the antioxidant enzyme activities. In this study, mice of TP supplemented group were administered TP 1 h before exhaustive exercise. In addition, the time to exhaustion of Ex + TP group mice were 263 ± 9.4 min.

Therefore, tissue sampling and blood collection immediately after the exhaustive exercise were approximately 320 min after the TP supplementation. It is possible that antioxidant ability of TP has not been reflected in the antioxidant ability because it had already been consumed or excreted during exercise. These results suggest that TP directly curbs the activities of some enzymes that generate ROS but does not change the antioxidant capacity. The activity of NADPH oxidase has been shown to be attenuated by the administration of polyphenols, and this enzyme could be potential target of TP^36–38^. Indeed, it cannot be excluded that Taheebo directly affects the ROS generation of NADPH oxidase and, this can explain the differences of redox biomarkers in the control and exercise groups. Hence, at rest the ROS production moderate, but during exhaustive exercise NADPH oxidase and xanthine oxidase are the main source of ROS, and the activities of these enzymes can be curbed by polyphenol supplementation^39–42^. At the present we don’t know much about the metabolites of TP which could enter into the bloodstream, therefore it cannot be completely excluded that some of them, if we have any, could have measurable effects on cellular signaling.

The results of Margaritelis *et al*.^43^ that subjects low exercise-induced oxidative stress exhibited the lowest improvements in a battery of classic adaptations to endurance training, indeed suggest an important role of redox signaling in exercise-associated adaptation. We fully agree with this data, which according to the current understanding is dose-dependent. Moderately elevated ROS seems to be beneficial, but high level of ROS can cause fatigue^44^. Exercise-induced ROS generation is intensity-dependent, aerobic exercise moderately while high intensity to exhaustion highly elevated ROS levels^33^. Therefore, Taheebo is more likely effective in high-intensity exercise by attenuating massive ROS production, than in low intensity exercise when the moderate increase of ROS might initiate beneficial adaptive response. Moreover, antioxidant treatment, including Taheebo, might curb the adaptive response of those who has low redox phenotype. Therefore, the antioxidant supplementation should be personalized.

Our data here suggest that Taheebo improves performance via metabolic changes (up-regulation of glycogen levels) and probably through the attenuation of oxidative stress.

Some studies have shown that long-termadministration of polyphenol or other plant extract increases endurance performance via mitochondrial biogenesis^45–47^. In this study, we measured gene expression of PGC-1α, a master regulator of skeletal muscle adaptation, and Cyto c, a marker of mitochondrial biogenesis. However, these gene expression levels did not change by TP supplementation. On the other hand, phosphorylation of AMPK and gene expression of SIRT1 were increased by TP administration. AMPK and SIRT1 are known as activators of PGC-1α. Single dose of TP supplementation increases the AMPK phosphorylation and gene expression level of SIRT1, but may be insufficient to change the gene expression level of PGC-1α or Cyto c.

**Figure 10:**
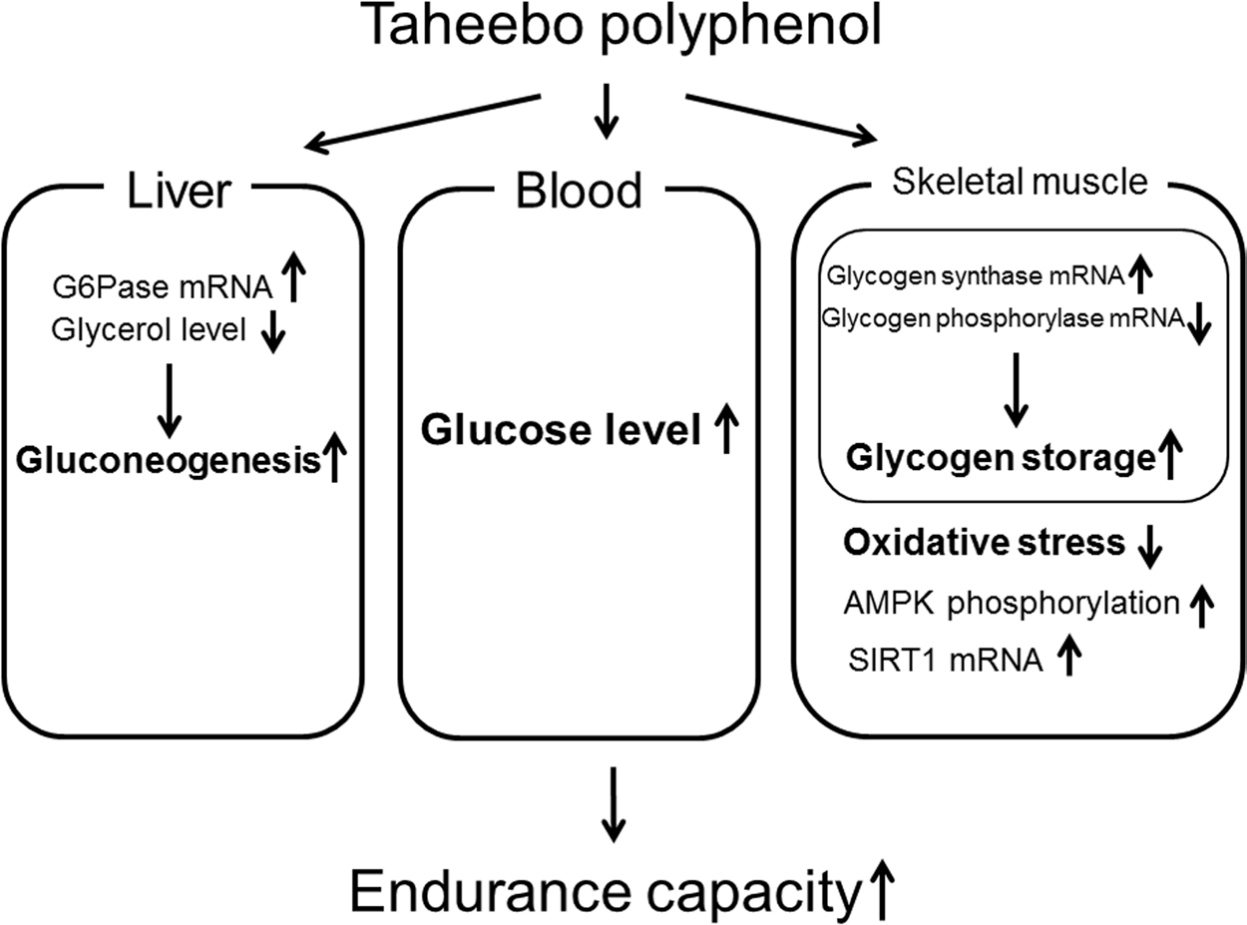
The suggested mechanisms by which Taheboo administration enhance exercise performance.

In conclusion, polyphenol fraction of taheebo improved endurance capacity of mice by acceleration of gluconeogenesis, increase of blood glucose level and up-regulation of glycogen content in skeletal muscle (Fig. 10). Moreover, TP decreased exercise-induced oxidative stress. Hence, TP can improve the endurance capacity via accumulation of muscle glycogen as well as attenuation of oxidative stress. Single dose administration of TP also increased phosphorylation of AMPK and gene expression level of SIRT1 but did not alter the marker of mitochondrial biogenesis.
